# In Situ Gelling Systems for Drug Delivery

**DOI:** 10.17795/jjnpp-20126

**Published:** 2014-06-01

**Authors:** Maryam Kouchak

**Affiliations:** 1 Nanotechnology Research Center, Department of Pharmaceutics, Faculty of Pharmacy, Ahvaz Jundishapur University of Medical Sciences, Ahvaz, IR Iran

**Keywords:** Phase Transition, Smart Polymers

In situ gelling systems are polymeric formulations that are in sol forms before entering in the body, but change to gel forms under the physiological conditions. The sol-gel transition depends on one or a combination of different stimuli, like pH change, temperature modulation, solvent exchange, ultra violet irradiation and the presence of specific ions or molecules. Drug delivery systems having such properties can be widely used for sustained delivery vehicle preparation of the bioactive molecules. Some important advantages of these smart systems are ease of application and reduced frequency of administration, as well as protection of drug from environmental condition changes. Various natural and synthetic polymers undergo in situ gel forming and potentially can be used for oral, buccal, rectal, vaginal, ocular, intraperitoneal and parenteral drug delivery. Pectin, xyloglucan, gellan gum, chitosan and alginic acid are some of the natural polymers (1). The pectin gelation occurs in the presence of calcium ions. When pectin is administered orally, divalent cations induce gel formation in the stomach (2). Xyloglucan exhibits thermally reversible gelation with body temperature and have been used for oral, ocular, rectal and peritoneal drug delivery. An oral liquid in situ gelling system could successfully produce sustained release formulation and solve quick transit of liquid preparations from the gastrointestinal tract (1). Dilute aqueous solutions of alginates form firm gels, on addition of di and trivalent metal ions. The formation of covalent bonds, leading to the perception of the insoluble cross linked alginate hydrogels, reduces the release of embodied drugs in alginate matrices. A formulation containing sodium alginate and calcium ions, being added to sodium citrate delays gelation process until the administered solution reaches the acidic environment of the stomach. Additionally, buccal adhesive in situ gels have been formulated, using ion or pH sensitive polymers, to prolong the contact time of antifungal drugs in the oral cavity (1). Ophthalmic in situ gels have been developed to prolong precorneal contact time of ocular drugs. Sodium alginate and hydroxypropyl methyl cellulose have been used in the in situ gel formulation of diclofenac ophthalmic delivery (3). In situ gel formation of gellan gum occurs due to temperature modulations or the cations induced. Temperature and ionic condition (Ca^2+^) in tear fluid cause a transition of aqueous solution of gellan into the gel state (4). Carbopol (poly acrylic acid) is a well-known pH dependent polymer, which stays in solution form at acidic pH but forms a low viscosity gel at alkaline pH. An in situ gel was formulated for ocular delivery of ofloxacin, using carbopol and hydroxypropyl methylcellulose (HPMC). The latter was used to impart the viscosity to the carbopol solution, while reducing its acidity (5). On the other hand, an aqueous solution of carbopol-HPMC system was designed and developed for plasmid DNA delivery (6). Pluronic F-127 is a triblock copolymer with nonionic nature, which undergoes in situ gelation by temperature change. It was used together with Carbopol 934 and HPMC to prepare a vaginal in situ gel incorporating clotrimazole-β-cyclodextrin complex to ensure long residence time of drug at the application site (7).

Chitosan aqueous solution forms a hydrated gel, like precipitate, at pH exceeding 6.2. Adding polyol salts, bearing a single anionic head, like glucose phosphate salts to chitosan aqueous solution can transform the cationic polysaccharides solution into thermally sensitive pH dependent gel. The sol form of such formulation (at the room temperature) turns into gel implants, when injected in vivo. This system was used successfully for tissue engineering applications (8). In situ gel formulations are of the most efficient treatment options, readily acceptable by physicians and patients. These systems offer successful controlled drug release and increase patient compliance.
